# Understanding patient health-seeking behaviour to optimise the uptake of cataract surgery in rural Kenya, Zambia and Uganda: findings from a multisite qualitative study

**DOI:** 10.1093/inthealth/ihab061

**Published:** 2021-09-28

**Authors:** Stevens Bechange, Emma Jolley, Patrick Tobi, Eunice Mailu, Juliet Sentongo, Titamenji Chulu, Maurice Abony, Moses Chege, Glenda Mulenga, Johnson Ngorok, Tesfaye Adera, Elena Schmidt

**Affiliations:** Sightsavers Zambia Country Office, 10247 Great East Road, Cresta Golf View, Villa 6, PO Box 37535, Lusaka 10101, Zambia; Sightsavers United Kingdom, 35 Perrymount Road, Haywards Heath, West Sussex, RH16 3BW, UK; Sightsavers United Kingdom, 35 Perrymount Road, Haywards Heath, West Sussex, RH16 3BW, UK; Department of Natural Sciences, Middlesex University, The Burroughs, London, NW4 4BT, UK; Sightsavers Kenya Country Office, Studio House, Ground Floor, Argwings Kodhek Road Hurlingham, PO Box 34690 00100 GPO, Nairobi, Kenya; Sightsavers Uganda Country Office, Second Floor, EADB Building, 4 Nile Avenue, PO Box 21249, Kampala, Uganda; Sightsavers Zambia Country Office, 10247 Great East Road, Cresta Golf View, Villa 6, PO Box 37535, Lusaka 10101, Zambia; Sightsavers Kenya Country Office, Studio House, Ground Floor, Argwings Kodhek Road Hurlingham, PO Box 34690 00100 GPO, Nairobi, Kenya; Sightsavers Kenya Country Office, Studio House, Ground Floor, Argwings Kodhek Road Hurlingham, PO Box 34690 00100 GPO, Nairobi, Kenya; Sightsavers Zambia Country Office, 10247 Great East Road, Cresta Golf View, Villa 6, PO Box 37535, Lusaka 10101, Zambia; Sightsavers Uganda Country Office, Second Floor, EADB Building, 4 Nile Avenue, PO Box 21249, Kampala, Uganda; Sightsavers United Kingdom, 35 Perrymount Road, Haywards Heath, West Sussex, RH16 3BW, UK; Sightsavers United Kingdom, 35 Perrymount Road, Haywards Heath, West Sussex, RH16 3BW, UK

**Keywords:** access to eye care, cataract surgery, health system, health-seeking behaviour, sub-Saharan Africa, uptake of services

## Abstract

**Background:**

Cataract is a major cause of visual impairment globally, affecting 15.2 million people who are blind, and another 78.8 million who have moderate or severe visual impairment. This study was designed to explore factors that influence the uptake of surgery offered to patients with operable cataract in a free-of-charge, community-based eye health programme.

**Methods:**

Focus group discussions and in-depth interviews were conducted with patients and healthcare providers in rural Zambia, Kenya and Uganda during 2018–2019. We identified participants using purposive sampling. Thematic analysis was conducted using a combination of an inductive and deductive team-based approach.

**Results:**

Participants consisted of 131 healthcare providers and 294 patients. Two-thirds of patients had been operated on for cataract. Two major themes emerged: (1) surgery enablers, including a desire to regain control of their lives, the positive testimonies of others, family support, as well as free surgery, medication and food; and (2) barriers to surgery, including cultural and social factors, as well as the inadequacies of the healthcare delivery system.

**Conclusions:**

Cultural, social and health system realities impact decisions made by patients about cataract surgery uptake. This study highlights the importance of demand segmentation and improving the quality of services, based on patients’ expectations and needs, as strategies for increasing cataract surgery uptake.

## Introduction

Cataract is a major cause of visual impairment globally, affecting 15.2 million people who are blind, and another 78.8 million who have moderate or severe visual impairment.^1^ While most cataracts cannot be prevented, cost-effective surgery can restore sight and significantly improve the quality of life and economic well-being of individuals and societies.^2,3^ However, the volume of cataract surgeries in many low- and middle-income countries (LMICs) remains low due to a complex interplay between supply-side (provider) and demand-side (patient) factors.^4^

In 2020, Mailu et al. published a systematic review of factors associated with the uptake of cataract surgery in LMICs^5^ that showed considerable variations between settings and study groups, from just 14% in Northern China to >91% in Southern India. The review identified several individual and contextual factors that influenced surgery uptake, but the number of papers was limited, and the studies were too heterogeneous to draw definitive conclusions across contexts. Among individual characteristics, the most consistent evidence was in relation to patients’ gender, age and visual acuity. With regard to context, the findings broadly supported an argument that the removal of user fees and moving services closer to the patient improved surgery uptake. However, the relationship was nuanced and dependent on local characteristics, such as population density, transport infrastructure and patients’ past experiences of services. The review highlighted examples of contexts where when even commonly known barriers, such as user fees and the cost of transport, had been removed, the uptake was not as high as one would expect. The authors called for a more in-depth understanding of patients’ eye health-seeking behaviour and demand for eye care in such contexts.

Studies of health-seeking behaviour more generally also draw attention to the complex nature of patient decision-making when it comes to the uptake of healthcare interventions.^6–8^ Balabanova et al. highlighted three broad groups of factors that intersected in shaping patient demand for services: (1) patients’ overall engagement with health and healthcare; (2) patient-provider relationships and previous experiences; and (3) the social context of decision-making.^9^ A few studies that have explored health-seeking behaviour in relation to cataract surgical services highlight several factors, such as a patient's perception of risk and severity of blindness,^10–12^ their family life and social relationships,^13–15^ as well as the perceived costs and benefits of surgery.^16^ However, overall, the evidence in this area remains weak, undermining opportunities for maximising the uptake of surgery and, subsequently, the efficiency of cataract services.

Against this background, we conducted a study that aimed to explore the factors influencing the uptake of surgery offered to patients with operable cataract, free at the point of use. The study was integrated in a large community-based eye care programme funded by UK Aid in three sub-Saharan Africa countries, namely, Kenya, Uganda and Zambia.^17^ In all three countries, patients were recruited through community outreach, offered free surgery and provided with transportation.

## Methods

### Study design and settings

We conducted a multisite qualitative exploratory study using key informant interviews (KIIs) and focus group discussions (FGDs). Data were collected during 2018–2019 in three districts of Zambia (Kalomo, Monze and Choma), two subregions of Uganda (Busoga and Karamoja) and three counties of Kenya (Turkana, Samburu and Laikipia). All of these settings are rural, and the main livelihood activity is subsistence agriculture, supplemented by small-scale trading in small townships and cattle-keeping for some sectors of the population. Access to healthcare is generally limited and travel distances are often lengthy over poorly maintained roads.

### Study participants and sampling

Study participants included patients with operable cataract and eye care service providers. The patient group included those who had been operated on and those who had either refused or could not access surgery for other reasons. Eye care providers included those directly involved in outreach and hospital activities. At each tertiary facility in each country, purposive sampling methods were used to identify the different health provider cadres, who were then invited to participate in the study.

Patients who participated in the study were recruited by purposive sampling from a list of 1778 patients diagnosed at outreach camps and referred for surgery in the 12 mo preceding the study (1 July 2016–30 June 2017). During the same period, 1496 patients were operated on. However, the uptake of surgery could not be accurately measured in these settings, as patients operated on for cataract included both walk-in and outreach patients, and only one-third of hospital and outreach records could be fully reconciled using patients’ names and places of residence.

All participants were selected to allow for a mix of gender, ages and locations. Participants were recruited until thematic saturation was reached. In total, 294 patients and 131 service providers were interviewed across three countries in 184 KIIs and 48 FGDs.

### Data collection procedures

Participants were contacted by telephone and invited for a face-to-face interview/FGD. The field team in each country included eight interviewers (four men and four women) led by a study supervisor (JS or SB in Uganda, EM in Kenya and TC or SB in Zambia). All the interviewers were local, spoke common local languages and were trained over a period of 1 wk on study ethics, interview techniques and data transcription. Topic guides were developed by the study team to address a broad set of aims related to health-seeking behaviour and the uptake of cataract surgery. Patients were asked about their reasons for taking up or not taking up surgery and how they made their decision. Those who took up the surgery were asked about their experiences and perception of services they received. Service providers were asked about cultural and community determinants of surgery uptake and the key challenges faced in the delivery of services.

Interviews with patients took place in their homes or the nearest community health centre. KIIs lasted up to 1 h; FGDs took 1.5–2.5 h. All KIIs and FGDs were conducted in the appropriate local language and were audio-recorded.

### Data analysis

Audio recordings were transcribed verbatim and translated into English. Transcript files were organised using NVIVO 12 (QSR International, Melbourne, Australia). Two team members (EM and SB) independently coded the transcripts. Data were analysed thematically, using a mix of inductive and deductive approaches. Themes and subthemes were identified based on the narrative content. This was followed by a data analysis workshop in each country and a combined workshop, which synthesised and compared findings across settings.

### Ethical considerations

All those patients who reported vision problems at the time of the interview were referred to a nearby health facility. FGD participants were provided with refreshments and their transport costs were reimbursed.

To ensure confidentially, quotations are only labelled with K=Kenya, U=Uganda, Z=Zambia and participant type/number.

## Results

### Participant characteristics

The characteristics of participants are summarised in Table 1. Participants represented a mix of ages, gender balance and a diverse range of patient and provider experiences.

**Table 1.** Characteristics of FGD and KII participants in rural Zambia, Kenya and Uganda (N=425)

		Zambia	Kenya	Uganda	Overall
**Patients**		**N=90**	**N=114**	**N=90**	**N=294**
Age in y, n (%)	41–50	4 (4)	3 (3)	7 (8)	14 (5)
	51–60	6 (7)	7 (6)	12 (13)	25 (9)
	61–70	49 (55)	74 (65)	48 (53)	171 (58)
	**≥**71	31 (34)	30 (26)	23 (26)	84 (28)
Gender, n (%)	Women	36 (40)	49 (43)	43 (48)	128 (43)
	Men	54 (60)	65 (57)	47 (52)	166 (57)
Marital status, n (%)	Single, separated/divorced	6 (7)	3 (3)	6 (7)	15 (5)
	Married	55 (61)	91 (80)	54 (60)	200 (68)
	Widowed	29 (32)	20 (17)	30 (33)	79 (27)
Highest level of education completed, n (%)	None	11 (13)	27 (24)	26 (29)	64 (22)
	Primary	74 (82)	68 (60)	60 (67)	202 (68)
	Post primary	4 (4)	13 (11)	3 (3)	20 (7)
	Tertiary	1 (1)	6 (5)	1 (1)	8(3)
Operated for cataract, n (%)	Yes	56 (62)	74 (65)	66 (73)	196 (66)
	No	34 (38)	40 (35)	24 (27)	98 (34)
**Healthcare providers**		**N=31**	**N=40**	**N=60**	**N=131**
Age in y, n (%)	**≤**30	18 (58)	24 (60)	36 (60)	78 (60)
	31–40	13 (42)	10 (25)	17 (28)	40 (30)
	**≥**41	0	6 (15)	7 (12)	13 (10)
Gender, n (%)	Women	7 (23)	16 (40)	19 (32)	42 (32)
	Men	24 (77)	24 (60)	41 (68)	89 (68)
Provider cadre, n (%)	Ophthalmologist	0	0	3 (5)	3 (2)
	Cataract surgeon	1 (3)	2 (5)	1 (2)	4 (3)
	OCO	6 (19)	8 (20)	16 (26)	30 (23)
	ON	4 (13)	4 (10)	4 (7)	12 (10)
	CHW	20 (65)	26 (65)	36 (60)	82 (62)

Abbreviations: CHW, community health worker; FGD, focus group discussion; KII, key informant interview; OCO, ophthalmic clinical officer; ON, ophthalmic nurse.

### Factors influencing cataract surgery uptake

For the purpose of this paper, themes emerging from the interviews have been organised into (1) surgery enablers and (2) barriers to surgery.

### Surgery enablers

Four reasons were given by patients in response to the question as to why they agreed to proceed with cataract surgery. First, patients wanted to regain control of their lives. Those whose sight was deteriorating feared losing their livelihood and thus their ability to look after their families:

*If I get blind…my children would not go to school, I can fail to feed them. Clothing and other necessary things would be impossible* (patient Z13, female).

Another operated-on patient commented:

*I had children and a wife to look after. Children needed to be in school…I had to save my eyes for them* (patient U38, male).

For these participants, this sense of ‘getting back control’ involved an ability to take decisions affecting their life, to restore order to the different aspects of their daily routines and family life and, consequently, to reduce their dependence on others. Another operated-on patient shared his experience:

*With good vision I can now move freely to visit friends, fence my home and cultivate the garden* (patient U11, male).

Second, many said that the positive testimonies of others, their ability to move and be independent, helped then to make a decision. Participants stressed that it was important for them to know that the services provided were of good quality, and that the healthcare workers were polite and treated patients with respect. One patient observed:

*One of them was my step-mother who could not see and was taken to Jinja; when she returned from there, she was very fine and could even go to the garden alone. She told us that the care was so good, […] the health workers were so friendly and treated her with respect. I felt reassured and decided to go [to the hospital] as well* (patient U46, male).

Third, the patient's family support played an important role in decision-making in all three settings. Patients said that their family members encouraged them to attend screening camps, accompanied them to hospital and provided care after the surgery:

*[M]y wife helped me to get on to on a motor bike and sit with me* (patient U22, male).

Finally, the majority of patients who had taken up the surgery offered said that their key motivator was free surgery, medication and food. Many were also happy about free transportation, which saved them time and money:

*Transport, we say thank you. From my side, I would not have gone to Namiyanga for I would not have managed to raise money for transport* (patient Z04, male).

### Barriers to surgery

Patients who had not been operated on included those who declined the surgery and those who could not access it for other reasons. The reasons given by these patients have been organised into 10 themes.

First, a number of patients across the three countries said that they did not feel the need or that the surgery would not improve much in their lives. For example, in Zambia, people believed that poor sight was natural for old people and, becuase old people were not economically active, there was no need to improve their vision. In Kenya, some patients thought that the restoration of their eyesight was pointless as they were old and were about to die anyway. In Uganda, there were participants who considered an eye problem as serious only if they were in pain or unable to see. A patient who had not been operated upon commented:

*I can't go to disturb doctors unless the eyes pain…if there is no pain, why should one come [to the hospital]! If the eyes pain or fail to see well, I will come* (patient U08, male).

Patients also had various superstitious beliefs about eyes. In Zambia, some communities believed that people go blind when they see a woman giving birth. In Kenya, eye diseases were associated with seeing water being poured into fire. Such patients preferred to see witchcraft doctors or to use traditional remedies:

*I have had an eye problem…as a result of witchcraft…when my husband died…I became blind, and I couldn't see anything* (patient K17, female).

*People blame witchcraft for their eye problems. They say probably their brother or neighbours have bewitched them because they have large herds of animals. Such beliefs make them disregard medical advice, even if you tell them, they have cataract which doesn't result from witchcraft* (healthcare provider K20, ophthalmic nurse).

Participants also described various myths and fears about cataract surgery. In all three countries, a number of people believed that the surgery was painful and that it could further damage their eyesight:

*I fear to be operated because I have seen many of them who were operated. Their eyes are damaged and shedding tears* (patient U28, female).

Some patients also believed that during surgery, the eyes got removed and replaced with the eyes of a goat or a chicken. In Kenya, the local word used for surgery was ‘scrapping’, which amplified scary tales about the procedure. In Zambia, patients were reluctant to travel in vehicles, as they feared they would be involved in an accident and die.

Patients who were willing but could not go to hospital described various challenges they faced. A common problem across the three settings was the lack of social support, specifically: (1) someone to accompany them to hospital; (2) someone to look after the house; and (3) someone to provide care after the surgery. In Kenya and Uganda, people from pastoralist communities repeatedly declined surgery because they did not have anyone to take care of their domestic animals, and they feared that others would steal their livestock. In Zambia, a number of patients feared being left alone in the house after surgery without access to food or medicine. This was found more often in the communities, where patients complained about the lack of surgery follow-up:

*Yes, there I had challenges because [I] thought…Who would nurse me after the surgery, who could be cooking for me?…I would not have managed it, dear* (patient Z32, female).

In all three countries, patients spoke about indirect costs associated with surgery. In Zambia, although patients knew that hospitals would provide them with food, some thought that it may not be sufficient. Others were concerned that the food would not be available to the relative who accompanied them. In Kenya, participants thought that they would have to purchase medicines that were not available in hospital.

In addition, in all three countries, some patients were concerned about the income they would lose while being in hospital. This was particularly common in the communities, where outreach camps were organised at the time of major farming activities:

*I'm the breadwinner of my family. If I go to hospital, my family will have nothing to eat…I'll persevere with the pain in my eyes, rather than my children…crying for food* (patient K08, female).

The role of gender in decision-making was highlighted in Kenya, where some men refused surgery because they were responsible for their families and did not want to become immobile. Women in this setting also declined surgery because of their perceived gender role, particularly in cases where their husband also needed surgery:

*For me I said I will not be operated, so that I will be able to give him [my husband] drugs,…I refused to be operated so that I could take care of him; I am the one who decided he will be operated first* (patient K19, female).

Both patients and healthcare providers pointed out that, in some settings, community mobilisation campaigns were limited in time and many people did not receive timely information or were misinformed about the time of surgeries:

*Mobilisation was not given enough time…[The programme] just made phone calls…we didn't have enough time to tell people about it* (healthcare provider Z13, community health worker).

*I went to that place twice. The first day, I was told to return on Thursday…Then we went, that's when I found…Thursday was the final day. They changed the day…So, they didn't attend to me* (patient Z22, male).

Participants also described situations where outreach camps had been cancelled but no one had informed the community, resulting in feelings of frustration and anger:

*What happened last year, they made an appointment to come at 08:00…people gathered…and we kept waiting. They never came; we just dispersed, and people did not eat anything...and this affected them so much* (patient Z16, male).

Another issue raised was insufficient quality of patient counselling. Although all healthcare providers agreed that the quality of information given to the patient was critical for the surgery uptake, they acknowledged that in many camps, healthcare workers were very busy, and the time for counselling was reduced to a minimum. Patients had no opportunities to ask questions or raise concerns. Many healthcare workers also said that they did not have adequate skills for patient counselling:

*Yes, there is a huge gap in the area of patient counselling…we are not counsellors. The programme should have included proper counsellors…people who are well trained* (healthcare provider U14, ophthalmologist).

*We give information, not counselling and also this is done in a group. It would be great to have a professional counsellor on the team* (healthcare provider U15, ophthalmic clinical officer).

Overall, many patients had little information about their treatment or what to expect from the hospital or after the surgery. In Kenya, some patients were told about the surgery only when they boarded the vehicle or arrived at the hospital. In Uganda, a female patient had expected to go home after the screening, but was invited to travel to hospital straightaway. She did not have any arrangements in place for her domestic duties and had to decline the offer. In Zambia, some patients did not have an opportunity to ask questions and felt that they were forced to sign a surgery consent form. In Kenya, a patient refused to sign a consent form because it indemnified the surgeon from any potential surgery complications:

*I refused because…on top of that card [consent form] they had written ‘If anything bad happens to your eyes after this operation, don't come complaining about it’. So, I saw this, and…[thought] maybe…these are not real doctors. That is how I refused* (patient K29, female).

For patients who attended hospitals, long queues and waiting times were a major challenge. This was particularly common in large districts, where limited numbers of healthcare staff had to serve very busy camps. In Kenya, hospital teams were so busy that patients were left unattended for many hours, with some deciding to go back home. In Uganda, healthcare workers reported losing patients who had undergone examination, pupil dilation and had signed consent, because there were no staff to attend to them. In those areas that had no local surgical teams, facilities relied on one visiting surgeon, who had many competing priorities, and many patients had to wait for a very long time. In some cases, the surgeon was reported as being reassigned to other duties and the surgeries were cancelled, leaving many patients disappointed and unhappy:

*At times, you come early, before they open the facility; but you will be made to wait and sometimes, you go back home without being treated* (patient Z31, male).

Also, in the areas where screening camps were organised over a long period of time, patients had to wait to be transported to the hospital for several days; and some reported that they had given up and changed their minds about the surgery.

Finally, the issue of staff skills and attitudes was raised in a number of interviews. Although most participants agreed that the staff conducting the screening were adequately skilled, there were some cases when patients were misdiagnosed and incorrectly referred for surgery. When such patients went to the hospital, they were re-examined and told that they did not qualify for surgery because their cataract was not mature or because they had another eye condition. In such cases, patients were upset and told others that the surgery had been denied to them.

In a few cases, staff were reported to be tired and irritated; and some patients complained about personal phone calls made by doctors or nurses during the examination:

*Sometimes, they have already started lighting you…then the phone rings and they start answering their phone. Sometimes, it's not even a phone call, they are just…checking…WhatsApp* (patient Z06, female).

*It depends on how patients are received from the camp…some nurses have bad attitude they can send back some patients due to patients’ poor hygiene* (healthcare provider U34, cataract surgeon).

## Discussion

Our study identified a number of individual and programme-level factors that influenced patients’ decisions about cataract surgery in the context where common barriers, such as user fees and the cost of transport, had been removed.

Contemporary theories of demand and supply^18,19^ suggest that different people have different needs and wants, which, in combination with purchasing power, constitute their demand for goods and services.^20,21^ It is therefore important to segment demand into discrete categories following a certain logic.^22^ Once the segmentation is complete, the supply side can be organised to deliver offerings to the chosen segments.^23^ Translating this to eye care, identifying different factors driving the demand for eye care services in different population subgroups is critical for shifting eye health production function and maximising the allocative efficiency of eye care services.^24^

In this study, approximately two-thirds of the patients interviewed were generally satisfied with the services they received. However, we identified patients who presented for eye examination but declined the surgery or were unable to proceed with it for other reasons. Some did not feel the need to improve their vision; for others, indirect costs or fears, competing household priorities or lost productivity, outweighed the potential benefits. It is important that managers of cataract programmes understand the size and profiles of these groups of patients and deploy behaviour-change strategies that can stimulate their demand.

Demand-side factors, such as personal and cultural beliefs, fears and the perceived benefits of services, have been documented previously.^25–27^ An interesting insight from this study is the evidence of intersection of capacity and quality of services with patients’ expectations and demands. Our findings show that well-organised and adequately resourced facilities with responsive staff and good communication result in a positive perception of services and stimulate surgery uptake. On the other hand, busy, overcrowded facilities, staffed with tired and overworked personnel, coupled with insufficient patient counselling and cancellation of surgeries, impact negatively on the perceived quality of care and deter patients. Our findings are in line with studies of customer satisfaction, which suggest that to stimulate demand, services should be reliable, responsive, assuring, empathetic and tangible in appearance of physical facilities and personnel.^28–30^

Our findings have important implications for eye care programmes. It may be true that many donor-supported programmes, which are usually limited in time and scale, may not be able to address deep-rooted cultural and social factors shaping patients’ engagement with health and healthcare. However, there are service-level factors, which are within the direct control of service providers and can be addressed within the scope of such programmes. Our study suggests that these are likely to include adequate human resources, timely community mobilisation campaigns, good quality patient counselling and effective communication between teams.

The multisite approach of our analysis helped to identify findings unique to specific contexts, while also highlighting broader patterns across settings.^31^ A key weakness was the limited individual level information on patient characteristics and insufficient linkages of outreach and hospital records. As a result, we were unable to segment patients ‘lost’ at different stages of the programme and to quantify the impact of these segments on the overall surgery uptake. It is important that future studies of eye health-seeking behaviour establish patient-tracking systems and engage patient quantitative and qualitative data in a complementary way.

In conclusion, this is one of a few studies that explore patients’ demands for cataract surgery delivered free at the point of use in rural sub-Saharan Africa settings. It highlights the importance of demand segmentation and improving the quality of services based on patients’ expectations and needs, as strategies for increasing cataract surgery uptake and maximising the value for limited resources available in eye care.

## Data Availability

Data are available upon reasonable request.
